# Multilevel analysis of factors associated with assistance during delivery in rural Nigeria: implications for reducing rural-urban inequity in skilled care at delivery

**DOI:** 10.1186/s12884-018-2074-9

**Published:** 2018-11-08

**Authors:** Bola Lukman Solanke, Semiu Adebayo Rahman

**Affiliations:** Department of Demography and Social Statistics, Faculty of Social Sciences, Obafemi Awolowo University, Ile-Ife, Nigeria

**Keywords:** Skilled assistance, Individual-level, Community-level, Delivery, Rural women, Nigeria

## Abstract

**Background:**

Studies have observed rural-urban inequity in the use of skilled delivery in Nigeria. A number of studies have explicitly examined associated factors of assistance during delivery in rural areas. However, the studies so far conducted in rural Nigeria have investigated mainly individual-level characteristics with near exclusion of community-level characteristics. Also, most of the studies that have investigated community-level influence on use of maternal healthcare services in Nigeria did not isolate rural areas for specific research attention. The objective of this study was to investigate the individual-level and community-level characteristics associated with assistance during delivery in rural Nigeria.

**Methods:**

The study analysed women data of 2013 Nigeria Demographic and Health Survey. A weighted sample size of 12,665 rural women was analysed. The outcome variable was assistance during delivery, dichotomised into ‘skilled assistance’ and ‘unskilled assistance’. The explanatory variables are selected individual-level characteristics (maternal education, parity, age at first birth, religion, healthcare decision, employment status, access to mass media, and means of transportation); and selected community-level characteristics (community literacy level, community childcare burden, proportion of women employed outside agriculture, proportion of women who perceived distance to facility as a big problem, community poverty level, and geographical region). The mixed-effects logistic regression was applied.

**Results:**

During the most recent deliveries, 23.0% of rural women utilised skilled assistance compared with 77.0% who utilised unskilled assistance. Maternal education, parity, religion, healthcare decision, access to mass media, and means of transportation were the individual-level characteristics that revealed significant effects on the likelihood of utilising skilled assistance during delivery, while community literacy level, community poverty level, community perception of distance to health facility, and geographic region were the community-level characteristics that revealed significant effects on the odds of using skilled assistance during delivery. Results of Intra-Class Correlation (ICC) supported significant community-level effects on the likelihood of using skilled assistance during delivery.

**Conclusions:**

Assistance during delivery is influenced by individual-level and community-level characteristics. Health policies and programmes seeking to reduce rural-urban inequity in skilled delivery should endeavour to identify and address important factors at both the individual and community levels of the social environment.

## Background

Rural areas in Nigeria have 51% population compared with 49% urban population [1], the rural areas also have higher incidences of pregnancies, child deliveries, and preventable maternal and child morbidities and mortality compared with the urban areas [2]. This makes the provision of maternal healthcare services across the continuum of maternal health care more compelling in the rural areas. However, evidence abounds that infrastructure for healthcare delivery particularly infrastructure for skilled care at delivery is markedly different between rural and urban areas, with the rural areas suffering infrastructural neglect in the country [3]. The existing rural health facilities in Nigeria are often bereft of adequate equipment, competent health personnel, and are largely inaccessible due to distance and poor road networks [2]. This situation which also exists in several sub-Saharan Africa countries often culminates not only in disparity in where rural and urban women deliver their babies [4], but also in disparity in the competency of persons providing assistance during delivery [5–7].

Assistance during delivery refers to the person attending child delivery. The person may be a ‘skilled attendant’ such as a doctor, nurse and midwife or an ‘unskilled attendant’ such as a traditional birth attendant, community extension worker, and friend/relative. The skills and competency of the person attending child delivery determine to a large extent whether complications during delivery are promptly identified and well-managed, as well as ensuring that hygienic practices are upheld during delivery [2]. While percentage of birth delivered by a skilled attendant in the urban areas of Nigeria increased from 65.4% in 2008 to 67.0% in 2013, the proportion in the rural areas of Nigeria, declined from 27.7% in 2008 to 22.7% in 2013 [8, 9]. This not only depict inequity in skilled care at delivery within Nigeria, it may continue to adversely affect birth outcome [10], exert negative impact on use of skilled delivery services by rural women [11], and may be a contributory factor to current poor maternal and newborn health in the country [2]. In addition, the situation of skilled delivery in Nigeria is a far cry from global expectation that all pregnant women irrespective of location be assisted by skilled attendants during delivery [12].

Health interventions in Nigeria such as the Midwives Service Scheme (MSS), which was established to improve human resources for health often, fail to reduce rural-urban disparity in skilled care at delivery due to challenges such as difficulty in retaining health personnel for rural health practice [13, 14]. The situation is aggravated by the unwillingness of many students undergoing medical training to take up future appointments in the rural and remote areas of the country [15]. Though, the World Health Organization (WHO) has made useful suggestions for recruiting and retaining rural health workforce [16], but achieving current and future human resource needs for sexual and reproductive health including maternal and child health in sub-Saharan Africa countries remains a huge challenge [17]. Thus, amid rural neglect and poor health delivery system, understanding the wide spectrum of factors affecting assistance during delivery remains relevant in reproductive health research in Nigeria.

Numerous studies across the world have confirmed that use of maternal healthcare services including assistance during delivery is mainly affected by a mixture of individual characteristics such as gender inequality [18, 19], maternal education [20], socio-economic inequality [21], spousal violence [22, 23], birth order and pregnancy wantedness [24], and community characteristics such as type of residence [25, 26], prevalence of larger family size [27], community education, community perception of distance to health facility, and community media saturation [28, 29]. Though, a number of studies have explicitly examined associated factors of assistance during delivery in rural areas [30–36], the studies so far conducted in rural Nigeria have investigated mainly individual-level characteristics with near exclusion of the community-level characteristics associated with assistance during delivery in rural areas [37–41]. Also, most of the studies that have investigated community-level influence on use of maternal healthcare services in Nigeria [28, 42–44] did not isolate rural areas for specific research attention.

This may undermine understanding the role of community-level characteristics (the characteristics of the social groups’ individual belong) in influencing utilisation of skilled care at delivery, and how community-level characteristics may contribute to developing effective community-based responses that could address existing inequity in utilisation of skilled care at delivery in the country. Community-level characteristics represent a unique social context that not only affect how individuals perceived and respond to health or other issues in the social environment, but also exert independent effects on the health outcomes of individuals in the community. Such independent effects made it possible for two women with identical socio-demographic and health characteristics to have different likelihood of using or not using skilled care at delivery. Thus, community-level characteristics may affect assistance during delivery by moderating how individuals in the community perceive the relevance and quality of assistance during child delivery. Increasing numbers of studies have provided research evidence that such social context is crucial for improving health behaviour [45–49].

The objective of the study was therefore, to examine the individual-level and community-level characteristics associated with assistance during delivery in rural Nigeria. This was with the view to providing additional policy-relevant information for addressing current rural-urban inequity in skilled care at delivery, and promoting equity in utilisation of skilled care at delivery in the country. The study was guided by the research question: to what extent are individual-level and community-level characteristics associated with assistance during delivery in rural Nigeria? The socio-ecological theory of health behaviour provides the theoretical underpinning of the study. The theory asserts that human health behaviour such as the use of maternal healthcare service is influenced at multiple levels within the social and physical environment such as the individual, household, community, societal and policy environment levels [50].

## Methods

### Study context

The study location is Nigeria, the most populous country in Africa [1]. Nigeria has a weak health delivery system that contributes to adverse maternal and child health outcomes [10, 51]. Also, the health delivery system in the country is inadequately funded [52]. Average national indices of maternal and child health, particularly utilisation of crucial maternal healthcare services such as skilled care delivery in the country is among the poorest in sub-Saharan Africa [2]. In the 5 years preceding the 2013 Nigeria Demographic and Health Survey (NDHS), among women who had a live birth, 61% received antenatal care from a skilled health provider; a lower proportion (51%) reported the recommended four or more antenatal care visits; 36% had facility-delivery, and 38% of deliveries were attended by a skilled health provider [9]. These show that utilisation of maternal healthcare services in the country need improvement. The Integrated Maternal, Newborn and Child Health (IMNCH) Strategy currently being implemented in the country seeks to boost utilisation of essential maternal care services across the continuum of maternal healthcare [2]. For instance, some of the IMNCH priority actions include increasing the coverage and quality of the Focused Antenatal Care; increasing demand for facility-based deliveries with skilled birth attendance; and ensuring that all mothers and newborns receive prompt postnatal check within 2 days. Nevertheless, the coverage of the IMNCH interventions is still very low particularly in rural and remote areas of the country [53]. The Federal Government has also made efforts to boost the funding of maternal and child health delivery in the country through The Subsidy Reinvestment and Empowerment Programme [51]. The programme has however recorded only marginal improvement in the situation of maternal, newborn and child health in the country, as drop out from the continuum of maternity care remain high in the country [54] with substantial rural-urban differentials in the use of maternal healthcare services [55].

Some health practitioners have commenced advocacy for free maternal and child health in the country to further boost utilisation of maternal healthcare services [56]. Some other health professionals have also advocated for the implementation of Conditional Cash Transfer (CCT) Scheme in the country, as another means of encouraging maternal healthcare use, particularly among socially disadvantaged women [57]. In spite of this efforts, negative perception of public health facilities may have continue to hinder improved utilisation among women in the country [58]. The Community-based health insurance programme, another initiative designed to enhance access to healthcare in the country is yet to successfully commence in many communities in the country [59]. These have provided compelling need for further investigation of associated factors of maternal healthcare use in the country.

### Data source and sample

This study was based on data collected from women of reproductive age in the 2013 NDHS. The 2013 NDHS is part of the series of cross-sectional Demographic and Health Survey (DHS) conducted across developing countries to provide reliable and internationally comparable information on the current state of fertility, childhood and adult mortality, family planning, and other sexual and reproductive health issues in developing countries. Relevant information about the design and implementation of the survey has been published [9]. In this study, only rural women were analysed. The study however, excluded women who were not currently married, and women who had no live birth in the 5 years preceding the survey. A weighted sample of 12,665 women were analysed in the study. A formal request to analyse the dataset was made to MEASURE DHS (the custodian of the DHS data) through online platform. Authorisation was granted.

### Outcome variable

The outcome variable in the study was assistance during delivery. This was a dichotomous variable with ‘skilled assistance’ and ‘unskilled assistance’ as categories. Skilled assistance in the study refers to delivery assistance provided by a doctor, nurse, midwife, or auxiliary nurse/midwife. This was based on the classification of skilled and unskilled health provider adopted in the 2013 NDHS [9]. But in some other countries, auxiliary nurse/midwife may not be classified as a skilled health provider [60]. Unskilled assistance in the study refers to delivery assistance provided by community extension worker, traditional birth attendants, friends/relatives, and no one. The category of interest in the study was the skilled assistance category.

### Explanatory and control variables

The explanatory variables in the study are individual-level and community-level characteristics. The individual-level characteristics analysed are maternal education, healthcare decision, parity, age at first birth, religion, employment status, access to mass media, and means of transportation. These characteristics were selected for analysis because previous studies have established their associations with utilisation of maternal healthcare services particularly in developing countries [20, 21, 23–26, 61–65]. Some of the variables were re-coded to suit the analytic framework of the study. Healthcare decision was based on whether women were involved in making decision about their own healthcare. Women who solely or jointly with male partner had final say on their healthcare decision were grouped as ‘participation’, while other women who had no say in the decision were grouped as ‘no participation’. Women’s access to the mass media was based on the frequency of listening to radio, watching television, or reading newspaper weekly. Women who did not listen to radio, watch television, or read newspaper during the week were grouped as ‘no access’, while those who listened to radio, watched television, or read newspaper at least once weekly were grouped as ‘moderate’ access. Women who listened to radio, watched television, or read newspaper more than once weekly were grouped as ‘high’ access. Women’s parity was divided into primiparity (one child ever born), multiparity (two to four children ever born), and grand multiparity (five or more children ever born).

Six community-level characteristics were analysed in the study. These are community childcare burden (proportion of women who had five or more children), community literacy level (proportion of women who cannot read and write at all), community poverty level (proportion of women in the poorest household quintile), proportion of women employed outside agricultural sector, community perception of distance to health facility (proportion of women who perceived distance to health facility as a big problem), and geographic region. The community-level characteristics were derived by aggregating the selected characteristic at the cluster level and then dividing into suitable categories. This was done because the variables are not directly available in the DHS data. Most studies that have analysed community-level variables using DHS data adopted the method [28, 29, 48]. In addition, to the selected individual-level and community-level variables, three variables were selected for statistical control. These are household wealth quintile, number of antenatal care visits, and timing of first antenatal care visits. The selection of these variables was guided by literature [24–27, 66].

### Data analysis

Three levels of statistical analyses were employed. The univariate analysis describes assistance during delivery using the pie chart, while respondents’ characteristics were presented using frequency distributions and percentages. At the bivariate level of analysis, cross tabulation of the research variables were carried out to show percentage of skilled assistance as changes in the categories of the explanatory variable occurs. This analytic level also describes the relationship between the variables using unadjusted binary logistic regression coefficient to reveal whether the relationships are positive or negative. Before the multivariate analysis was carried out, a Variance Inflation Factor (VIF) was calculated to detect extent of multi-collinearity of the independent variables using the mean VIF score. This was needed to determine the suitability of the selected variables for multivariate analysis. As a rule of thumb in regression analysis, a mean VIF score of less than 5 is tolerated, while a mean VIF score of 5–10 suggests that the regression coefficients might be inadequately estimated [67, 68]. At the multivariate level, the mixed-effects logistic regression was used to determine extent of variation in the use of skilled assistance attributable to individual-level and community-level characteristics. The mixed-effects logistic regression model consists of two parts, namely, the fixed effect and the random effect [43]. The model was specified as:

$$ \log \left(\frac{\pi_{ij}}{1-{\pi}_{ij}}\right)={\beta}_0+{\beta}_1{x}_{ij}+{\beta}_2{x}_{2 ij}+\dots +{\beta}_8{x}_{8 ij}+{\beta}_9{z}_{1j}+{\beta}_{10}{z}_{2j}+\dots {\beta}_{14}{z}_{6j}+{\mu}_{0j} $$

Where:

*π*_*ij*_ is the log of odds of skilled assistance.

(1 − *π*_*ij*_) is the log of unskilled assistance.

x and z are the explanatory variables for the likelihood of skilled assistance.

x_1_ to x_8_ are the individual-level characteristics.

z_1_ to z_6_ are the community-level characteristics.

*β*_0_is the overall intercept.

*β*_1_…*β*_14_ are the regression coefficients for the explanatory variables x_1_ to x_8_, and z_1_ to z_6_.

*u*_0*j*_ is the community-level random effect (assumed to be normally distributed with mean equal to 0 and variance equal to ${\sigma}_{\mu {0}^2}$).

The fixed effects were estimated using odds ratio of adjusted binary logistic regression, while the random effects were estimated using the Intra-Class Correlation (ICC) calculated as: $\frac{\tau }{\tau +\left[\frac{\pi^2}{3}\right]}$where *τ* is the estimated community-level variance [43]. The ICC ranges from 0 to 1, with ICC of 1 indicating that women in the community have identical use of skilled assistance during delivery, and with ICC of 0 indicating that women in the community do not have identical use of skilled assistance in the community. Four models were fitted in the study. Model 1 included only individual-level characteristics, while Model 2 was based solely on community-level characteristics. Model 3 included both individual-level and community-level characteristics. Model 4 was the full model that included the explanatory and control variables. The models were fitted using the *xtmelogit* command of Stata version 12 [69]. Model adequacy was examined using the Wald chi-square which assesses the statistical significance of the model. The 5% alpha level was considered statistically significant. Analyses were performed using Stata version 12.

## Results

### Univariate results

**Fig. 1:**
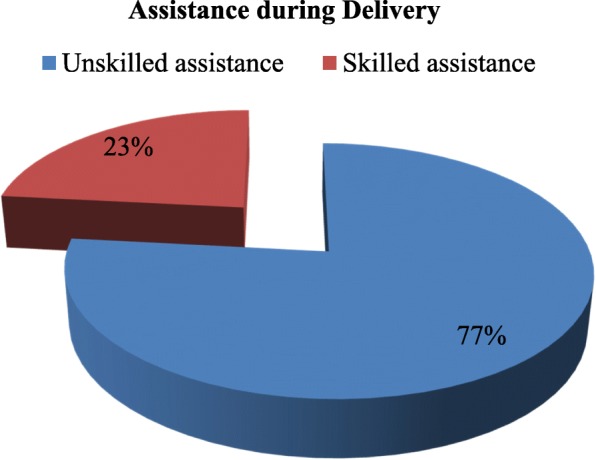
Distribution of assistance during Delivery among women, Rural Nigeria

Figure 1 presents distribution of respondents by assistance during delivery. As shown in the figure, during their most recent deliveries, slightly less than a quarter of respondents (23.0%) utilised ‘skilled assistance’ while the majority of respondents (77.0%) utilised ‘unskilled assistance’. Table 1 presents respondents’ socio-demographic profile. Nearly two-thirds of the respondents’ had no formal education. However, among respondents with educational attainments, primary education was dominant. The majority of respondents were either multiparous (42.7%) or grand multiparous (41.7%) women. Reproductive age interval of 15–19 years was the dominant age interval at first birth among the respondents. The proportion of the respondents who had first birth at age twenty-five or older ages was less than one-tenth of the respondents. The majority of the respondents were employed during the survey. However, slightly more than one-third of the respondents were unemployed during the survey. Muslim women compared with women practicing Christianity or other religions were dominant among respondents.

**Table 1:** Respondents socio-demographic characteristics, rural Nigeria, 2013

Characteristic	Number of women (*n* = 12,665)	Percentage
Maternal Education		
None	8100	64.0
Primary	2294	18.1
Secondary	1992	15.7
Higher	278	2.2
Parity		
Primiparity	1.979	15.6
Multiparity	5411	42.7
Grandmultiparity	5275	41.7
Age at first birth (years)		
14 or less	1248	9.8
15–19	7405	58.5
20–24	2999	23.7
25 or older	1013	8.0
Employment status		
Unemployed	4361	34.4
Employed	8304	65.6
Religion		
Christianity	3608	28.5
Islam	8816	69.6
Others	241	1.9
Healthcare decision		
No Participation	9139	72.2
Participate	3526	27.8
Access to mass media		
No access	5654	44.6
Moderate access	2860	22.6
High access	4151	32.8
Means of Transportation		
None/animal cart	5108	40.3
Boat/canoe	210	1.7
Bicycle	1710	13.5
Motorcycle/scooter	5064	40.0
Car/truck	573	4.5
Household wealth quintile		
Poorest	4400	34.7
Poorer	3937	31.1
Middle	2560	20.2
Richer	1306	10.3
Richest	462	3.7
Number of antenatal care visit		
No Visit	6279	49.5
1–3 Visits	1629	12.9
4 or more visits	4756	37.6
Timing of first antenatal visit		
1st Trimester	1831	14.5
2nd Trimester	4508	35.6
3rd Trimester	6326	49.9
Community childcare burden		
Low	4602	36.3
Medium	4934	39.0
High	3129	24.7
Community literacy level		
Low	3552	28.0
Medium	4522	35.7
High	4591	36.3
Community poverty level		
Low	3761	29.7
Medium	4237	33.4
High	4667	36.9
Proportion employed outside agriculture		
Low	4120	32.5
Medium	4156	32.8
High	4389	34.7
Proportion who perceived distance as a big problem		
Low	6556	51.8
High	6109	48.2
Geographic Region		
Northern region	10,303	81.4
Southern region	2362	18.6

Source: Author’s analysis based on 2013 Nigeria Demographic and Health Survey

More than two-thirds of the respondents did not participate in their healthcare decision. Slightly more than two-fifths of respondents had no access to the mass media. However, nearly one-third of the women had ‘high’ access to the mass media. More than two-fifths of respondents either had no modern means of transportation or had animal-drawn cart as means of transportation. Motorcycle/scooter was the dominant modern means of transportation available to the respondents who reported a modern means of transportation. Higher proportions of the respondents were in the poorest (34.7%) and poorer (31.1%) wealth quintiles compared with the respondents in other wealth categories. Nearly half of the respondents (49.5%) had no antenatal care visits prior to their most recent deliveries, while nearly half of respondents reported some numbers of antenatal care visits. However, more than one-third of the respondents (37.6%) reported the recommended four or more antenatal care visits prior to their most recent deliveries. Distribution of respondents by timing of first antenatal care visits however revealed that nearly half of the respondents had their first antenatal care visit in the third trimester of pregnancy, while slightly more than one-third of respondents (35.6%) had their first antenatal care visit in the second trimester of pregnancy.

More than one-third of the respondents live either in communities with low (36.3%) or moderate (39.0%) childcare burden that is communities with low or medium proportion of high parous women but nearly one-quarter of the respondents live in communities with high childcare burden. In contrast, more than one-third of the respondents live in communities with either medium or high proportions of women who cannot read or write at all. Likewise, the distribution of respondents by community poverty level showed that the proportions of women who live in communities with either medium or high poverty level were more than one-third of respondents. The proportions of respondents employed outside agriculture were similar in the communities, though with slightly higher proportion of respondents living in communities with high proportion of women employed outside agriculture. More respondents live in communities where more than half of the women perceived distance to health facility as a big problem. Women from the northern region of the country were dominant among the respondents.

### Bivariate results

Table 2 presents proportions of skilled assistance and the bivariate relationships between the research variables. Maternal education and assistance during delivery were positively and significantly related with consistent increase in the proportion of skilled assistance as maternal education improves from one lower level to the next higher level. Parity and assistance during delivery were negatively associated. As maternal parity increased from primiparity (one-CEB) to multiparity (4-CEB), and to grand multiparity (5 or more CEB), proportion of skilled assistance respectively decreased from 29.9 to 25.3%, and 18.9%. Age at first birth and assistance during delivery are positively and significantly related with the highest proportion of skilled assistance among women who had their first birth at age 25 years or older ages (45.0%). Likewise, employment status was positively and significantly associated with assistance during delivery (β = 0.591; CI: 0.411–0.770). Employed women had higher skilled assistance compared with unemployed women (26.7% vs. 16.8%).

**Table 2:** Proportion of skilled assistance and unadjusted binary logistic regression coefficients showing bivariate relationships, Rural Nigeria, 2013

Characteristic	% of Skilled assistance	Coeff.	95% CI
Maternal education			
None^RC^	9.1	–	–
Primary	35.0	1.684*	1.476–1.891
Secondary	58.8	2.658*	2.450–2.866
Higher	87.0	4.205*	3.728–4.683
Parity			
Primiparity^RC^	29.9	–	–
Multiparity	25.3	−0.230**	−0.370, −0.091
Grandmultiparity	18.9	−0.604*	−0.746, −0.462
Age at first birth (years)			
14 or less^RC^	14.6	–	–
15–19	17.7	0.232**	0.036–0.428
20–24	33.5	1.080*	0.865–1.294
25 +	45.0	1.566*	1.278–1.855
Employment status			
Unemployed^RC^	16.8	–	–
Employed	26.7	0.591*	0.411–0.770
Religion			
Christianity^RC^	51.3	–	–
Islam	11.9	−2.053*	−2.280, −1.825
Others	20.8	−1.388*	−2.002, −0.774
Healthcare decision			
No participation^RC^	16.3	–	–
Participation	41.4	1.287*	1.124–1.451
Access to mass media			
No access^RC^	12.9	–	–
Moderate access	20.5	0.556*	0.341–0.770
High access	39.5	1.487*	1.298–1.676
Means of transportation			
None/animal cart^RC^	21.6	–	–
Boat/canoe	15.2	−0.430	−0.955, 0.094
Bicycle	15.3	−0.423**	−0.682, − 0.164
Motorcycle/scooter	25.4	0.211**	0.030–0.391
Car/truck	47.0	1.167*	0.887–1.447
Household wealth quintile			
Poorest	4.8	–	–
Poorer	16.1	1.333*	1.081–1.586
Middle	38.4	2.509*	2.235–2.782
Richer	58.3	3.317*	3.011–3.622
Richest	78.2	4.258*	3.834–4.682
Number of antenatal care visits			
No visit	5.6	–	–
1–3 visits	22.4	1.583*	1.352–1.814
4 or more visits	47.1	2.711*	2.496–2.925
Timing of first antenatal care visit			
1st trimester	51.9	–	–
2nd trimester	37.7	−0.576*	−0.745, −0.408
3rd trimester	4.8	−3.066*	−3.297, −2.835
Community childcare burden			
Low	36.1	–	–
Medium	16.5	−1.051*	−1.398, −0.704
High	15.2	−1.150*	−1.524, −0.776
Community Literacy Level			
Low	56.5	–	–
Medium	17.0	−1.850*	−2.128, − 1.573
High	3.9	−3.462*	−3.772, − 3.153
Community Poverty Level			
Low	52.9	–	–
Medium	18.4	−1.606*	− 1.903, − 1.309
High	3.9	−3.315*	−3.627, − 3.004
Proportion employed outside agriculture			
Low	22.0	–	–
Medium	23.3	0.074	−0.286, 0.434
High	24.7	0.152	−0.216, 0.520
Proportion who perceived distance as a big problem			
Low	13.1	–	–
High	34.3	1.244*	0.936–1.552
Geographic region			
Northern region	14.8	–	–
Southern region	60.6	2.180*	1.891–2.470

Notes: Coeff. (Coefficient), RC (Reference Category), *p < 0.01, **p < 0.05

Religion was negatively associated with assistance during delivery. Christian women compared with Muslim and other women had higher prevalence of skilled assistance. The relationship between healthcare decision and assistance during delivery was significantly positive (β = 1.287; CI: 1.124–1.451) with higher prevalence of skilled assistance among women who participated in the decision compared with women who did not participate in the decision (41.4% vs. 16.3%). Access to mass media and assistance during delivery were significantly positively related. As access to mass media improve from ‘none’ to ‘moderate’ and ‘high’, the proportion of skilled delivery increased from 12.9 to 20.5%, and 39.5% respectively. Means of transportation had mixed relationship with assistance during delivery. The relationship was negative among women whose means of transportation were boat/canoe or bicycle but positive among women whose means of transportation were motorcycle/scooter or car/truck. However, the proportion of skilled assistance was highest among women whose means of transportation were car or truck (47.0%).

Household wealth quintile had significant positive relationship with assistance during delivery. As wealth quintile improved from one lower wealth group to another higher wealth group, the proportion of skilled assistance progressively improved. For instance, proportion of skilled assistance improved from 38.4% among women in ‘middle’ wealth group to 58.3% among women in ‘richer’ wealth group. Antenatal care visits relates positively with assistance during delivery. The proportion of skilled assistance was 5.6% among women who had no antenatal care visit but increased to 22.4% among women who had 1–3 antenatal care visits. It further increased to 47.1% among women who had the recommended four or more antenatal care visits. Timing of first antenatal care visit was negatively associated with assistance during delivery with lowest proportion of skilled assistance (4.8%) among women whose first antenatal care visits took place in the third trimester of pregnancy compared with 51.9% of skilled assistance among women whose first antenatal care visit took place within the first trimester of pregnancy.

Community childcare burden was negatively associated with assistance during delivery with reduction in the proportions of skilled assistance as childcare burden increases in the communities. The relationship between community literacy level and assistance during delivery was also negative with lowest proportion of skilled assistance (3.9%) among women in communities with high proportion of women who cannot read or write at all. Community poverty level was negatively associated with assistance during delivery. The proportion of skilled assistance was highest among women who live in communities with low proportion of women in the poorest wealth group. Proportion of women employed outside agricultural sector relates positively with assistance during delivery. The proportion of skilled assistance increased as the proportion of women employed outside agriculture improved. For instance, the proportion of skilled assistance was higher among women who live in communities with high proportion of women employed outside agricultural sector compared with women in communities with low proportion of women employed outside agriculture (24.7% vs. 22.0%). Community perception of distance to health facility was positively associated with assistance during delivery with lower proportion of skilled assistance (13.1%) among women who live in communities with low perception of distance to health facility as a big problem to accessing healthcare. Likewise, geographic region and assistance during delivery were significantly positively related (β = 2.180; CI: 1.891–2.470). Southern women compared with their northern counterpart had higher use of skilled assistance (60.6% vs. 14.8%).

### Multivariate results

Prior to fitting the multivariate models, the VIF computed revealed a mean VIF score of 3.36. This not only confirms that the explanatory variables were not significantly multi-collinear, it also suggest that the variables were sufficient for adequate estimation of the regression coefficients. The variables were then included in the mixed-effects analyses. Table 3 presents the fixed-effects on the likelihood of skilled assistance during delivery. The Wald chi-square confirm that all the fitted models were statistically significant (*p* < 0.01). In Model 1 (Wald χ^2^ = 288.3; p < 0.01), six individual-level characteristics, namely, maternal education, parity, religion, healthcare decision, access to mass media, and means of transportation had significant effects on the likelihood of skilled assistance. For instance, grand multiparous women were 48.2% less likely to utilise skilled assistance compared with primiparous women (OR = 0.518: CI: 0.420–0.640). In contrast, women who participated in healthcare decision were 43.5% more likely to utilise skilled assistance compared with women who did not participate in healthcare decision (OR = 1.435; CI: 1.215–1.693).

**Table 3:** Odds ratio of adjusted binary logistic regression showing fixed effects on the likelihood of skilled assistance

Characteristic predicting skilled assistance	Model 1 (Wald χ^2^ = 288.3; *p* < 0.001)		Model 2 (Wald χ^2^ = 333.4; *p* < 0.001)		Model 3 (Wald χ^2^ = 367.9; *p* < 0.001)		Model 4 (Wald χ^2^ = 474.9; *p* < 0.001)	
	Odds Ratio	95% CI	Odds Ratio	95% CI	Odds Ratio	95% CI	Odds Ratio	95% CI
Maternal education								
None^RC^	–	–			–	–	–	–
Primary	1.994*	1.623–2.450			1.481*	1.215–1.806	1.205	0.996–1.458
Secondary	3839*	2.583–5.706			2.703*	2.120–3.447	1.840*	1.464–2.313
Higher	4.131*	3.184–5.358			13.114*	7.533–22.831	5.793*	3.474–9.660
Parity								
Primiparity^RC^	–	–			–	–	–	–
Multiparity	0.538*	0.440–0.657			0.532*	0.437–0.648	0.575*	0.476–0.695
Grand multiparity	0.518*	0.420–0.640			0.506*	0.411–0.624	0.564*	0.462–0.689
Age at first marriage (years)								
14 years or less^RC^	–	–			–	–	–	–
15–19 years	0.843	0.654–1.086			0.839	0.654–1.077	0.811	0.635–1.035
20–24 years	1.104	0.840–1.453			1.044	0.798–1.366	0.981	0.755–1.275
25 years or older	1.121	0.800–1.571			1.033	0.743–1.437	0.945	0.685–1.304
Employment Status								
Unemployed^RC^	–	–			–	–	–	–
Unemployment	1.169	0.991–1.378			1.094	0.931–1.286	1.033	0.883–1.208
Religion								
Christianity^RC^	–	–			–	–	–	–
Islam	0.317*	0.237–0.423			0.741**	0.560–0.982	0.750**	0.577–0.974
Traditional/others	0.354**	0.188–0.664			0.558	0.299–1.040	0.712	0.390–1.300
Healthcare Decision								
No Participation^RC^	–	–			–	–	–	–
Participation	1.435*	1.215–1.693			1.290**	1.100–1.513	1.213**	1.041–1.413
Access to mass media								
None^RC^	–	–			–	–	–	–
Moderate	1.138	0.932–1.388			1.039	0.856–1.260	0.934	0.774–1.127
High	1.817*	1.506–2.193			1.562*	1.304–1.871	1.255**	1.055–1.494
Means of Transportation								
None/animal cart^RC^	–	–			–	–	–	–
Boat/canoe	0.513**	0.293–0.900			0.386*	0.226–0.659	0.392*	0.234–0.659
Bicycle	0.966	0.748–1.249			1.028	0.800–1.321	1.052	0.824–1.341
Motorcycle/scooter	1.425*	1.207–1.683			1.383*	1.177–1.626	1.222**	1.046–1.429
Car/truck	2.169*	1.566–3.003			1.984*	1.447–2.719	1.438**	1.055–1.960
Community childcare burden								
Low^RC^			–	–	–	–	–	–
Medium			0.886	0.623–1.260	1.071	0.759–1.511	1.079	0.796–1.462
High			0.752	0.505–1.116	0.936	0.635–1.380	0.978	0.694–1.378
Community literacy level								
Low^RC^			–	–	–	–	–	–
Medium			0.208*	0.136–0.319	0.389*	0.256–0.591	0.489*	0.339–0.706
High			0.072*	0.040–0.128	0.170*	0.095–0.302	0.346*	0.208–0.575
Community poverty level								
Low^RC^			–	–	–	–	–	–
Medium			0.382*	0.252–0.579	0.447*	0.299–0.670	0.602**	0.421–0.860
High			0.159*	0.090–0.282	0.213*	0.122–0.370	0.402*	0.241–0.670
Proportion employed outside agriculture								
Low^RC^			–	–	–	–	–	–
Medium			1.065	0.747–1.518	1.064	0.749–1.511	0.932	0.683–1.274
High			1.416	0.982–2.043	1.331	0.923–1.919	1.084	0.783–1.502
Proportion who perceived distance as big problem								
Low^RC^			–	–	–	–	–	–
High			2.774*	1.999–3.850	2.433*	1.770–3.345	1.815*	1.374–2.397
Geographic region								
Northern^RC^			–	–	–	–	–	–
Southern			2.103*	1.399–3.162	1.750**	1.163–2.634	1.818**	1.265–2.611
Household wealth quintile								
Poorest^RC^							–	–
Poorer							1.261	0.990–1.606
Middle							1.630**	1.227–2.166
Richer							1.819**	1.296–2.552
Richest							2.834*	1.761–4.560
Number of antenatal care visits								
None^RC^							–	–
1–3							1.430	0.991–2.064
4+							2.602*	1.803–3.756
Timing of first antenatal visit								
1st trimester^RC^							–	–
2nd trimester							0.802**	0.680–0.946
3rd trimester							0.252*	0.173–0.369

Notes: *p < 0.001, **p < 0.05

In Model 2 (Wald χ^2^ = 333.4; *p* < 0.01), four community-level characteristics, namely, community literacy level, community poverty level, community perception of distance to health facility, and geographic region revealed significant independent effects on the likelihood of skilled assistance. For instance, women in communities with high proportion of women who cannot read or write at all were less likely to utilise skilled assistance compared with women in communities with low proportion of women who cannot read or write at all (OR = 0.072; CI: 0.040–0.128). Likewise, women in communities with high proportion of women in poorest wealth group were less likely to utilise skilled assistance compared with women in communities with low proportion of women in poorest wealth group (OR = 0.159; CI: 0.090–0.282). The inclusion of both individual-level and community-level characteristics in Model 3 (Wald χ^2^ = 367.9; *p* < 0.01) reinforced the statistical significance of the individual-level and community-level characteristics which showed significant effects in the earlier models.

In the full model (Wald χ^2^ = 474.9; p < 0.01), the inclusion of the three control variables did not result in any substantial change in the pattern of the fixed effects on assistance during delivery. In the model, the likelihood of utilising skilled assistance increased significantly as maternal education improved. For instance, women who attained higher education were nearly six times more likely to use skilled assistance compared with uneducated women (OR = 5.793: CI: 3.474–9.660). Likewise, multiparous women (OR = 0.575; CI: 0.476–0.695) and grand multiparous women (OR = 0.564; CI: 0.462–0.689) were less likely to utilise skilled assistance compared with primiparous women. Also, Muslim women were 25% less likely to utilise skilled assistance compared with Christian women (OR = 0.750; CI: 0.577–0.974). Women who participated in healthcare decision were 21.3% more likely to utilise skilled assistance compared with women who did not participate in healthcare decision (OR = 1.213; CI: 1.041–1.413). Access to mass media revealed significant effect on the odds of skilled assistance. Women who had high access to the mass media were 25.5% more likely to utilise skilled assistance compared with women in the reference category (OR = 1.255; CI: 1.055–1.494). Similarly, women who had car/truck were 43.8% more likely to use skilled assistance compared with women who had no means of transportation (OR = 1.438; CI: 1.055–1.960).

Community literacy level exerted significant influence on the likelihood of using skilled assistance. Women who live in communities with high proportion of women who cannot read or write at all were 65.4% less likely to utilise skilled assistance compared with women in communities with low proportion of women who cannot read or write at all (OR = 0.346; CI: 0.208–0.575). Also, community poverty level revealed significant influence on the odds of skilled assistance. Women in communities with high proportion of women in poorest wealth group were 59.8% less likely to use skilled assistance during delivery compared with women in communities with low proportion of women in poorest wealth group (OR = 0.402; CI: 0.241–0.670). Women in communities where high proportion of women perceived distance to health facility as a big problem were more likely to use skilled assistance during delivery compared with women in the reference category (OR = 1.815; CI: 1.374–2.397). Women in southern region of Nigeria were almost twice more likely to use skilled assistance compared with women in northern region of the country (OR = 1.818; CI: 1.265–2.611). The three selected control variables showed significant influence on the likelihood of using skilled assistance during delivery. The odds of using skilled assistance during delivery increased progressively as household wealth group improved. Women who had four or more antenatal care visits were almost three times more likely to utilise skilled assistance during delivery compared with women who had no antenatal care visits (OR = 2.602; CI: 1.803–3.756). Women whose first antenatal visit took place either in the second or third trimester were less likely to use skilled assistance during delivery compared with women whose first antenatal visit took place in the first trimester of pregnancy.

Table 4 presents the random effects of the mixed-effects models. As shown in the table and using the significance of the LR test, all the models fitted showed a good fit. In the absence of individual-level and community-level characteristics, the result of the ICC in the empty model reveal that the use of skilled assistance was highly identical among women in the community (ICC = 0.705). When only individual variables were included in Model 1, the ICC reduced to 0.515 to indicate that higher proportion of variation in the use of skilled assistance was attributable to community-level characteristics (ICC = 0.515). This proportion further reduces to 0.381 in Model 2. With the combination of individual-level and community-level characteristics in Model 3, the variation in the use of skilled assistance attributable to community-level characteristics remained substantial (ICC = 0.359) indicating that more than one-third of the women in the communities had identical use of skilled assistance during delivery. In the full model, more than a quarter of variation in the use of skilled assistance during delivery were attributable to community-level characteristics (ICC = 0.286).

**Table 4:** Random Effects showing influence of community characteristics on use of skilled assistance during delivery

Parameter	Empty Model	Model 1	Model 2	Model 3	Model 4
Community level variance (SE)	7.850 (0.925)	3.488(0.449)	2.023 (0.258)	1.840 (0.243)	1.320 (0.177)
Log likelihood	− 5028	− 4698.3	− 4761.8	− 4550.8	− 4229.6
LR test	χ^2^ = 4192.2; *p* < 0.001	χ^2^ = 1231.1; *p* < 0.001	χ^2^ = 1024.3; *p* < 0.001	χ^2^ = 781.2; p < 0.001	χ^2^ = 571.9; *p* < 0.001
ICC	0.705	0.515	0.381	0.359	0.286

## Discussion

This study examined individual-level and community-level characteristics associated with assistance during delivery in rural Nigeria. It improves research attention on skilled delivery in the rural areas which in spite of poor health infrastructure is usually the area with higher incidences of pregnancies and child deliveries in Nigeria [2, 8, 9]. The study not only compliment existing studies that have explicitly examined associated factors of assistance during delivery in rural areas [30–33], it also analysed community-level influence on the use of assistance during delivery in rural areas of Nigeria compared with previous rural studies that investigated mainly individual-level characteristics [34–38]. The study provided additional support for the socio-ecological model [50] by confirming the significance of factors operating at both individual and community levels, and thus consistent with previous studies that stressed the importance of community factors in initiatives to improve human health behaviour particularly health-seeking behaviour [45–49]. Based on this finding, it is plausible to assert that factors affecting utilisation of skilled assistance during delivery in rural areas of Nigeria operates at multiple levels, which require that initiatives seeking to boost utilisation of skilled assistance during delivery should endeavour to identify and address the important factors at each distinct level of the social environment.

The study found that utilisation of skilled assistance at delivery is very poor in the rural areas of Nigeria. Though, the 23.0% prevalence of skilled assistance found in the study was higher than the 13.0% reported in an earlier study in Nigeria [53], it corroborates prevalence reported in both the 2008 and 2013 NDHS [8, 9]. There are two possible reasons for poor utilisation of skilled assistance at delivery in rural Nigeria. The first reason may relate to poor health infrastructure in rural areas of the country which made unskilled health providers more accessible to rural women [2]. In many rural and remote areas of the country, there are no Primary Health Care (PHC) centres, and where the PHCs are available, many of the health personnel including skilled birth attendants are usually unwilling to reside in the rural communities [13]. The implementation of the MSS was expected to improve maternity care in the rural areas of Nigeria, but it is yet to yield significant positive results due to challenges such as retention of health personnel in rural areas, availability and training of midwives, and lack of political will by States and Local government in the country [14]. Also, where PHCs are available, several adjoining communities may have to depend on its services. This usually creates distance barrier to some of the communities and may affect utilisation. This implies that utilisation of skilled delivery may likely improve in the rural areas if more health centres are provided with qualified health personnel. As evident in the study, though a high proportion of the women perceived distance to health facility as a big problem, but they reported a higher use of skilled assistance than the proportion who perceived distance to health facility as not a big problem which indicates that distance to health facility may not be as important as the availability of health facilities and accessibility to desired services. It is therefore important that government at all tiers of the Nigerian federation should improve public investment on the provision of more health facilities for rural dwellers. In the interim, the staff strength at existing rural health facilities could be expanded by the recruitment of more qualified health personnel especially those already trained in delivery care.

The second reason that may account for poor use of skilled assistance among rural women is the socio-demographic condition of the women. As found in the study, educational attainment was poor among the women, the majority of them were multiparous, had no autonomy on their healthcare, and belong to poorest household wealth group. Such social conditions have been found in earlier studies [2, 51, 53] to promote non-use of maternal healthcare services. Beyond the provision of healthcare centres in rural areas, it is imperative that more concerted efforts be made to improve the socio-economic conditions of rural women. This could be achieved through more rural development efforts such as the creation of more roads to enhance economic activities of rural women, and strengthening existing rural empowerment programmes. The advocacy for free Maternal and Child Health (MCH) services [56] should be given fresh impetus in the country to encourage more States of the federation to implement free MCH services particularly for rural families. This will reduce the economic burden of healthcare among rural women. Where free MCH services are not feasible, rural women could be assisted to improve their use of skilled delivery through the design and implementation of more CCT programme already piloted in some States of the country [57].

Hence, individual characteristics of women cannot be ignored in interventions to improve utilisation of skilled assistance during delivery. Existing initiatives should endeavour to focus more on three specific characteristics, namely, education, autonomy on healthcare decision, and religion. Education is central to all efforts aiming to improve use of skilled delivery in the rural areas because in spite of the effectiveness of existing interventions, utilisation may remain low in the absence of widespread awareness of the dangers of unskilled delivery. Also, several socio-cultural practices such as unequal power relations between male and female partners, and lack of male involvement in maternal healthcare [19] that undermine prompt health-seeking behaviour are best confronted through the provision of accurate information, communication and education on maternal and child health issues. It is thus important that the current IMNCH strategy reposition its information and communication initiative through massive public health education messages using the mass media particularly the radio which are more widely used in the rural areas.

This should be complimented by promoting women autonomy in the communities. Men in the communities should be mobilised through public campaigns to encourage women to have sole autonomy on issues affecting their reproductive life. Also, there should be more interventions to focus on Muslim women. As evident in this study and consistent with findings in similar previous studies [63, 65], there is unequal likelihood of maternal healthcare services utilisation among Christian and Muslim women. This may indicate that the different religions have different practices that impact women’s perception and use of services. This differences in religious beliefs as it relates to maternal health should not only be fully understood by health policy planners, it is also important that religion be made an important component of future maternal healthcare interventions in line with the submission of several scholars on the subject [61, 62, 64, 70].

The study further found that community-level characteristics are crucial for improvement of the use of skilled assistance at delivery in the rural areas. This necessitates more community-based maternal healthcare interventions in rural Nigeria to increase the coverage of the existing community-based programme of the IMNCH Strategy [2]. Community-based interventions are more important in the rural areas because women in the rural communities tend to have identical perception and use of accessible health services. Findings from the study provided evidence that community-level factors such as community poverty or literacy levels have important effects on use of skilled assistance during delivery. Hence, existing community-based intervention such as the community extension workers programme [2] needs to be strengthened in the country. Finally, the antenatal care programme should be appropriately perceived as a veritable means of reaching rural women about other services within the continuum of maternal and child healthcare. In many rural communities and as found in this study, high proportions of women had no antenatal care visits. Also, among those who reported antenatal care visits, more than one-third of the visits took place in the third trimester of pregnancy. Such visitations may not be as a result of seeking adequate antenatal information and counselling, but more likely to be a response to complications or health challenges. Thus, there should be renewed efforts to strengthen the antenatal care programme not only in terms of expanding its coverage to all rural communities to enhance visits in the first trimester of pregnancy, but to also ensure that women who visited health facility for antenatal care are followed up, retained in the continuum to ensure facility deliveries and use of skilled attendants during delivery, and ultimately to reduce rate of drop out from maternity care [31, 54].

This study cannot claim to have established a cause-effect relationship between the explanatory and outcome variables of the study due to the cross-sectional nature of the data analysed. The study however buttress existing observation that both individual-level and community-level characteristics have important implications for the use of maternal healthcare services among women. Likewise, the socio-ecological theory could not be applied in its original form because the data analysed did not provide sufficient information required to capture all its theoretical constructs. It is not impossible that a different pattern of relationship between the variables may be observed if all the theoretical constructs are derivable. Future studies on the subject matter may therefore consider the use of primary data to enhance availability of all needed information.

## Conclusions

This study provided additional empirical evidence that utilisation of maternal healthcare services particularly skilled delivery service is influenced by individual-level characteristics such as maternal education, parity, religion, and healthcare decision, and community-level characteristics such as community poverty level, and community literacy level. The study provided evidence that to a significant extent, individual-level and community-level characteristics are associated with assistance during delivery in rural Nigeria. Initiatives seeking to reduce rural-urban inequity in skilled delivery should endeavour to identify and address the important factors at the individual and community levels of the social environment.

## Abbreviations

DHS: Demographic and Health Survey
FMoH: Federal Ministry of Health
MSS: Midwives Service Scheme
NDHS: Nigeria Demographic and Health Survey
PRB: Population Reference Bureau
WHO: World Health Organization
